# Health in Food Systems Policies in India: A Document Review

**DOI:** 10.34172/ijhpm.2021.18

**Published:** 2021-03-15

**Authors:** Adithya Pradyumna, Arima Mishra, Jürg Utzinger, Mirko S. Winkler

**Affiliations:** ^1^Swiss Tropical and Public Health Institute, Basel, Switzerland.; ^2^University of Basel, Basel, Switzerland.; ^3^Azim Premji University, Bengaluru, India.

**Keywords:** Agriculture, Food Systems, India, Intersectoral Coordination, Nutrition, Policy Analysis

## Abstract

**Background:** Food systems affect nutritional and other health outcomes. Recent literature from India has described policy aspects addressing nutritional implications of specific foods (eg, fruits, vegetables, and trans-fats), and identified opportunities to tackle the double burden of malnutrition. This paper attempts to deepen the understanding on how health concerns and the role of the health sector are addressed across food systems policies in India.

**Methods:** This qualitative study used two approaches; namely (i) the framework method and (ii) manifest content analysis, to investigate national-level policy documents from relevant sectors (ie, food security, agriculture, biodiversity, food processing, trade, and waste management, besides health and nutrition). The documents were selected purposively. The textual data were coded and compared, from which themes were identified, described, and interpreted. Additionally, mentions of various health concerns and of the health ministry in the included documents were recorded and collated.

**Results:** A total of 35 policy documents were included in the analysis. A variety of health concerns spanning nutritional, communicable and non-communicable diseases (NCDs) were mentioned. Undernutrition received specific attention even beyond nutrition policies. Only few policies mentioned NCDs, infectious diseases, and injuries. Governing and advisory bodies were instituted by 17 of the analysed policies (eg, food safety, agriculture, and food processing), and often included representation from the health ministry (9 of the 17 identified inter-ministerial bodies).

**Conclusion:** We found some evidence of concern for health, and inclusion of health ministry in food policy documents in India. The ongoing and planned intersectoral coordination to tackle undernutrition could inform actions to address other relevant but currently underappreciated concerns such as NCDs. Our study demonstrated a method for analysis of health consideration and intersectoral coordination in food policy documents, which could be applied to studies in other settings and policy domains.

## Background

**Key Messages**

** Implications for policy makers**

- While the disproportionate policy focus on undernutrition is justified, there is a need to also emphasise other health implications of food systems, such as obesity, non-communicable diseases (NCDs), and agrarian distress.
- Dietary guidelines have been created for the Indian population by government-affiliated nutrition research institutions, but these have not been adequately used to guide food policy.
- Representation from the Ministry of Health and Family Welfare (MoHFW) was listed in inter-ministerial regulatory, advisory and executive groups of other food-related ministries. While this is encouraging, there is a need for further clarity on whether such representation is influencing food systems to become more health-sensitive.
- The intersectoral approaches being used and planned for undernutrition can provide a template for addressing other food systems-related health concerns. This would also be relevant towards fulfilling the National Health Policy (NHP) goal of the “Health in All” approach.

** Implications for the public**

Our research identified that undernutrition is a priority concern for various ministries in India. Several measures have been implemented to address vulnerability to undernutrition among marginalised communities, women, and children. Detailed insights are also available for measures towards food safety. However, several other relevant health concerns, such as obesity, cardiovascular disease (CVD), agricultural injuries, agrarian distress, and hazards of food wastes were not adequately addressed, despite being critical population health concerns. In our view, though the health ministry is often represented in inter-ministerial groups to govern food systems, measures to systematically include health considerations in food policy should be explicitly described and further enhanced.

A United Nations report highlighted that “Food systems are at the heart of the 2030 Agenda for Sustainable Development.”^1^ Food systems include “*all the elements (environment, people, inputs, processes, infrastructures, institutions, etc ) and activities that relate to the production, processing, distribution, preparation and consumption of food, and the outputs of these activities, including socio-economic and environmental outcomes*.”^2^ These elements of food systems involve various actors, each of whom pursues different goals and priorities.^3,4^ For instance, farmers are concerned about sustained income, and the public health sector aims to address malnutrition.^3^ One important outcome of food systems is people’s health and well-being,^3^ and thus it warrants specific attention. The challenge, however, is that the linkages between food systems and health are manifold and complex.^5,6^

 Food systems-related health concerns include malnutrition (undernutrition and obesity),^7^ non-communicable diseases (NCDs; eg, diabetes, cardiovascular disease [CVD], and cancer),^8^ antibiotic resistance (due to indiscriminate use particularly in the livestock sector),^9^ vector-borne diseases (VBDs; due to increased risk of malaria from water resources and deforestation),^10,11^ parasitic infections,^12^ exposure to pesticides,^13^ and farmers’ suicides.^14^ In addition, due to greenhouse gas emissions from food systems, climate change-related health risks such as extreme weather events, malnutrition, and VBDs are enhanced.^11,15^ Globally, undernutrition and dietary risks are currently the top risk factors for mortality.^8^ There is a growing recognition that addressing challenges related to nutrition and NCDs need strong intersectoral coordination.^16,17^

 In that context, impacts of food systems policies on public health are being examined internationally.^17,18^ The role of governments towards healthy food environments for NCD prevention was seen as regulatory (ie, legal frameworks, economic instruments, and guidelines) and catalyst (ie, awareness creation, building multi-stakeholder partnerships, and making funding available for coordinated action).^19^ Financial and technical assistance towards production of healthier food was reported to have strong potential from a health perspective.^20^ However, the general challenge towards intersectoral coordination for better health has persisted over decades, which may be driven by the complexity of setting up and managing such a system, its potential to compromise the existing political economy, and importantly, the neglect of preventive measures even within the health sector.^21^ In situations where systems for intersectoral coordination have been instituted, the impact on health equity was reported to be lower for upstream interventions as compared to downstream interventions.^22^

 Historically, since independence in 1947, the focus of food systems-related policy in India has mainly been on food security. The early focus was on famine prevention, which also included food-for-work programmes.^23^ Subsequently, safety nets for food security and welfare were enhanced,^24^ and greater thrust was provided for maternal and child nutrition.^25^ However, India is not on track to meet the Global Targets 2025 on stunting, exclusive breast feeding, and anaemia.^26^ In addition, risk factors for NCDs are of increasing concern, with one in five adults aged above 30 years having high systolic blood pressure, and 7.7% of adults aged above 20 years showing elevated fasting sugar levels.^27^ The proportion of individuals who are overweight has increased rapidly between 2006 and 2016.^28^ Overall, undernutrition and dietary risks were the first and third highest ranked risk factors for mortality in India.^29^ Meanwhile, agrarian distress, driven by low productivity, poor remuneration, and indebtedness, is also of concern, overtly manifesting through farmers’ suicides.^30^ Hence, there is a considerable burden of health impacts directly associated with food systems in India, warranting a closer look at how policies are addressing them. It has been suggested that the entire range of food systems policies should be considered to improve health outcomes.^31^ The policy space in India has been analysed for opportunities to address malnutrition, NCDs,^4^ and the consumption of fats,^32^ trans-fats,^33^ and fruits and vegetables.^34^ Nutrition, despite being the most obvious health outcome of food systems, was perceived as a minor consideration by food supply policy actors in India,^4^ which is a matter of concern.

 Nutrition is but one of many health outcomes of food systems policies, as discussed earlier. The review of literature did not reveal any studies that have addressed whether and how various relevant health concerns have been considered in food policies in India. Moreover, besides health aspects of food consumption policies and pathways, those related to food production are important in India as the majority of the population is dependent on agriculture for livelihood.^35^ Finally, the recent National Health Policy (NHP) indicated the need for a “Health in All” approach,^36^ but the inclusion in health policies of intersectoral coordination with food systems actors, and the role of the health ministry in food systems governance in India have not been adequately described in the literature.

 This paper attempts to deepen the understanding on how health, including nutrition and several other related health concerns, and the role of the health sector, specifically the Ministry of Health and Family Welfare (MoHFW) and other central health institutions, are included and addressed across food systems-related policy documents in India. The following research questions governed the current analysis: First, what were the health concerns addressed in food systems policies in India? Second, whether and how these concerns were addressed in health policies themselves? Third, what were the roles identified for the health ministry in food systems governance in India? While the first two questions helped us to better understand the consideration for health outcomes, the last question looked at governance avenues for these considerations.

## Methods

###  Study Design

 A qualitative study design was employed, using two approaches to content analysis; namely (*i*) the framework method, as described by Gale and colleagues,^37^ and (*ii*) manifest content analysis, as described by Hsieh and colleagues.^38^ The framework method is a type of thematic analysis that facilitates the identification of relevant themes to describe the findings.^37^ This method was used because the breadth of study topics was large, stemming from diverse sectors. This approach also helped contextualise data from individual policies within the pool of analysed policies.^37^ Manifest content analysis was used to document mentions of specific diseases or risks, and health-related and other institutions.^38^

###  Data Sources

 National-level policy documents, including statutory, technical, or official documents, relevant to broad food systems activities and outcomes,^1^ such as agriculture, food security, food processing, trade, agro-biodiversity, health, nutrition, and NCDs were employed. The analysis was initiated with 14 purposively selected documents, which were identified by the first author based on prior knowledge and relevant literature.^4^ The documents were downloaded in English language from governmental portals to ensure authenticity.^39^ This sample was enhanced, first with other policy documents that were cited in the initially analysed set, second by referring back to the food systems definition for aspects that were not adequately covered by the current sample, and third, based on data gaps in the analytical framework (eg, documents on vegetable production and food waste management). Table provides the list of policy documents analysed in the current paper. Of the 35 analysed policies, 17 were ‘agricultural,’ seven were ‘social,’ two were ‘environmental,’ two were ‘trade,’ and one was ‘industrial.’ Six ‘health’ policies were also included to better understand the health ministry perspective on food systems governance and relevant health concerns.

**Table:** Food Systems Policies From India (2001 to 2018) Included in the Current Analysis

**Name of Policy (Abbreviation)**	**Year**	**Type of Document**	**Sector**
PPVFRA 2001^40^	2001	Statutory	Agriculture
BDA 2002^41^	2002	Statutory	Environment
PDS (part of the X^th^ Plan document)^42^	2002	Technical report	Social; intersectoral
FSSA 2006^43^	2006	Statutory	Health; intersectoral
ADWDRS 2008^44^	2008	Official (scheme)	Agriculture
NPF 2007^45^	2008	Official (policy)	Agriculture; intersectoral
NPCDCS 2008^46^ and 2013^47^	2008	Official (programme)	Health
MNREGS (2008 guidelines)^48^	2008	Official (based on statute)	Social; intersectoral
Common Guidelines for WSD Projects 2008 (revised 2011)^49^	2011	Official (policy)	Agriculture; intersectoral
VIUC 2011 guidelines^50^	2011	Official (scheme)	Agriculture
NMAET (XII^th^ plan, 2012)^51^	2012	Official (scheme)	Agriculture
NFSM (part of the XII^th^ plan document)^52^	2012	Official (scheme)	Agriculture; intersectoral
NFS (2013 guidelines)^53^	2013	Official (scheme)	Agriculture; intersectoral
NMFP (2013 guidelines)^54^	2013	Official (scheme)	Industry; intersectoral
NFSA 2013^55^	2013	Statutory	Social; intersectoral
The NPC 2013^56^	2013	Official (policy)	Social; intersectoral
MIDH 2014^57^	2014	Official (scheme)	Agriculture
NMOOP 2014^58^	2014	Official (scheme)	Agriculture
NMSA 2014^59^	2014	Official (scheme)	Agriculture
NPMCR 2014^60^	2014	Official (policy)	Agriculture
FP 2015^61^	2015	Official (policy)	Agriculture
FTP 2015-2020^62^	2015	Official (policy)	Trade
NLM (2016 guidelines)^63^	2016	Official (scheme)	Agriculture
SWMR 2016^64^	2016	Statutory	Environment; intersectoral
NAPCCHH 2016^65^	2016	Official (policy)	Health; intersectoral
RKVY (14^th^ Financial Commission 2017)^66^	2017	Official (scheme)	Agriculture
Draft PMB 2017^67^	2017	Statute (draft)	Agriculture; intersectoral
NHP 2017^36^	2017	Official (policy)	Health
ICDS (2017 guidelines)^68^	2017	Official (scheme)	Health; intersectoral
NNS 2017^28^	2017	Official (policy)	Health; intersectoral
ARMPD 2017^69^	2017	Technical report	Social; intersectoral
FDIP 2017^70^	2017	Official (policy)	Trade
ESCB 2017-2018^71^	2018	Technical report	Social; intersectoral
MEFSB 2018^72^	2018	Technical report	Social; intersectoral
PSSG^73^	-	Official (scheme)	Agriculture

Abbreviations: ADWDRS, Agricultural Debt Waiver and Debt Relief Scheme; ARMPD, Annual Report of Ministry of Public Distribution; BDA, Biological Diversity Act; ESCB, Economic Survey Chapter 1 (Part of Budget); FDIP, Foreign Direct Investment Policy; FP, Fertilizer Policy; FSSA, Food Safety and Standards Act; FTP, Foreign Trade Policy; ICDS, Integrated Child Development Services; MEFSB, Macroeconomic framework statement for Budget; MIDH, Mission for Integrated Development of Horticulture; MNREGS, Mahatma Gandhi National Rural Employment Guarantee Scheme; NAPCCHH, National Action Plan for Climate Change and Human Health; NFS, Nutri-farms Scheme; NFSA, National Food Security Act; NFSM, National Food Security Mission; NHP, National Health Policy; NLM, National Livestock Mission; NMAET, National Mission on Agricultural Extension and Technology; NMFP, National Mission on Food Processing; NMOOP, National Mission on Oilseed and Oil Palm; NMSA, National Mission on Sustainable Agriculture; NNS, National Nutrition Strategy; NPC, National Policy for Children; NPCDCS, National Programme for the Prevention and Control of Cancer, Diabetes, Cardiovascular Diseases and Stroke; NPF, National Policy for Farmers; NPMCR, National Policy for Management of Crop Residues; PDS, Public Distribution System; PMB, Pesticide Management Bill; PPVFRA, Protection of Plant Varieties and Farmers Rights Act; PSSG, Price Support Scheme guidelines; RKVY, Rashtriya Krishi Vikas Yojna; SWMR, Solid Waste Management Rules; VIUC, Vegetable Initiative for Urban Clusters; WSD, Watershed Development.

###  Analysis

 For the thematic analysis by the framework method, aspects that were of interest based on the study objectives and relevant literature were: (*i*) health and nutrition outcomes addressed; (*ii*) healthy diets and dietary diversity; (*iii*) social protection and equitable access for nutritional security; (*iv*) trade and investment for healthy food systems; (*v*) environmental sustainability of food systems; (*vi*) health system role in food systems-related health action; and (*vii*) intersectoral coordination. The coding scheme was guided by these interests (deductive) and also the data themselves (inductive) (the scheme is presented in Supplementary file 1, Table S1). The working analytical framework was developed and applied to all the policy documents using the OpenCode 4.03 software.^74^

 Summarised data (with meaning of the original data, and references to relevant quotations) from each data item were charted into a spread-sheet containing the analytical framework.^75^ Interpretation was done by understanding the content and source of data within each category of the analytical framework, and identifying potential synergies and differences between data from various sectoral and policy documents. Analytical memos were prepared for themes that addressed the main research questions.^37,76^

 The manifest content analysis was conducted to document (*i*) the various diseases and health risks, and (*ii*) the various ministries and health institutions mentioned in each document. For this, during the reading and re-reading of the documents, notes were made about the specific diseases, risks and ministries that were mentioned in the document. In addition, as a second check, the following generic terms were searched for using the search function of the PDF reader: ‘health,’ ‘risk,’ ‘wellbeing,’ ‘disease,’ ‘nutrition,’ and ‘ministry.’ These frequencies were tabulated and presented as figures using Microsoft Excel 2016^75^ and R statistical software version 3.5.1.^77^ Further details are available under each figure.

## Results

 The findings have been presented under the five themes that helped describe the data: (*i*) ‘Undernutrition recognised and addressed as a cross-sectoral concern;’ (*ii*) ‘The focus on NCDs;’ (*iii*) ‘Supporting healthy and balanced diets;’ (*iv*) ‘Addressing health concerns beyond nutrition and NCDs;’ and (*v*) ‘Involvement of health ministries in food systems governance.’ The relevant policies contributing to each specific finding is cited in the text using their respective abbreviations from Table. Findings from the manifest content analysis have been reported using figures, interspersed within the appropriate themes. The first paragraph under each theme summarises the findings from the theme.

###  Undernutrition Recognised and Addressed as a Cross-sectoral Concern

 Nutrition was identified as an important policy concern, with aspects related to nutrition mentioned or discussed in several policies (FSSA, ICDS, NAPCCHH, NHP, NNS, NPCDCS, MIDH, NFS, NFSA, NFSM, NLM, NPF, and RKVY). The focus was primarily on undernutrition (ICDS, NFS, NFSA, and NNS). Three policies were dedicated almost exclusively to undernutrition (ICDS, NFS, and NNS). Moreover, undernutrition was the only health concern to have an agricultural scheme dedicated to it (NFS). Several groups were deemed vulnerable to undernutrition from a social security perspective (ie, challenges in accessing food; ARMPD, NFSA, NPF, and PDS) and a public health perspective (ie, high prevalence of undernutrition; ICDS, NAPCCHH, and NNS). The factors articulated as contributing to vulnerability were poverty (ARMPD, NFSA, and NLM), age (ICDS, NAPCCHH, NFSA, NNS, and NPC), gender (ICDS, NAPCCHH, NFSA, NHP, and NNS), infirmity (ARMPD, ICDS, NFSA, and NPF), caste (NNS), disasters or shocks (NAPCCHH, NNS, and PDS), and remoteness of residence (NFSA and PDS). Towards addressing undernutrition and the associated vulnerability, the approaches mentioned included provision of food grains (NFSA), food supplementation for target groups (ARMPD, ICDS, NFSA, NPC, and PDS), healthcare support (ICDS, NHP, and NNS), and broader societal interventions related to empowering and enabling vulnerable communities (NAPCCHH, NLM, NNS, and NPF).

*“Malnutrition in India, especially among children and women, is widespread, acute and even alarming” *(NFS).

 Micronutrient deficiencies and its consequences such as restriction of growth and development were other aspects of undernutrition that received attention (NHP). This included deficiencies of iron, vitamin A, zinc, iodine (NNS), calcium, selenium, and magnesium (NFS). Serious consequences of undernutrition were recognised in health policies (NNS and NHP) and also one agricultural policy (NFS).

*“The consequences, in terms of malnutrition and health, are devastating and can result in blindness, stunting, disease and even death” *(NFS).

**Figure 1:**
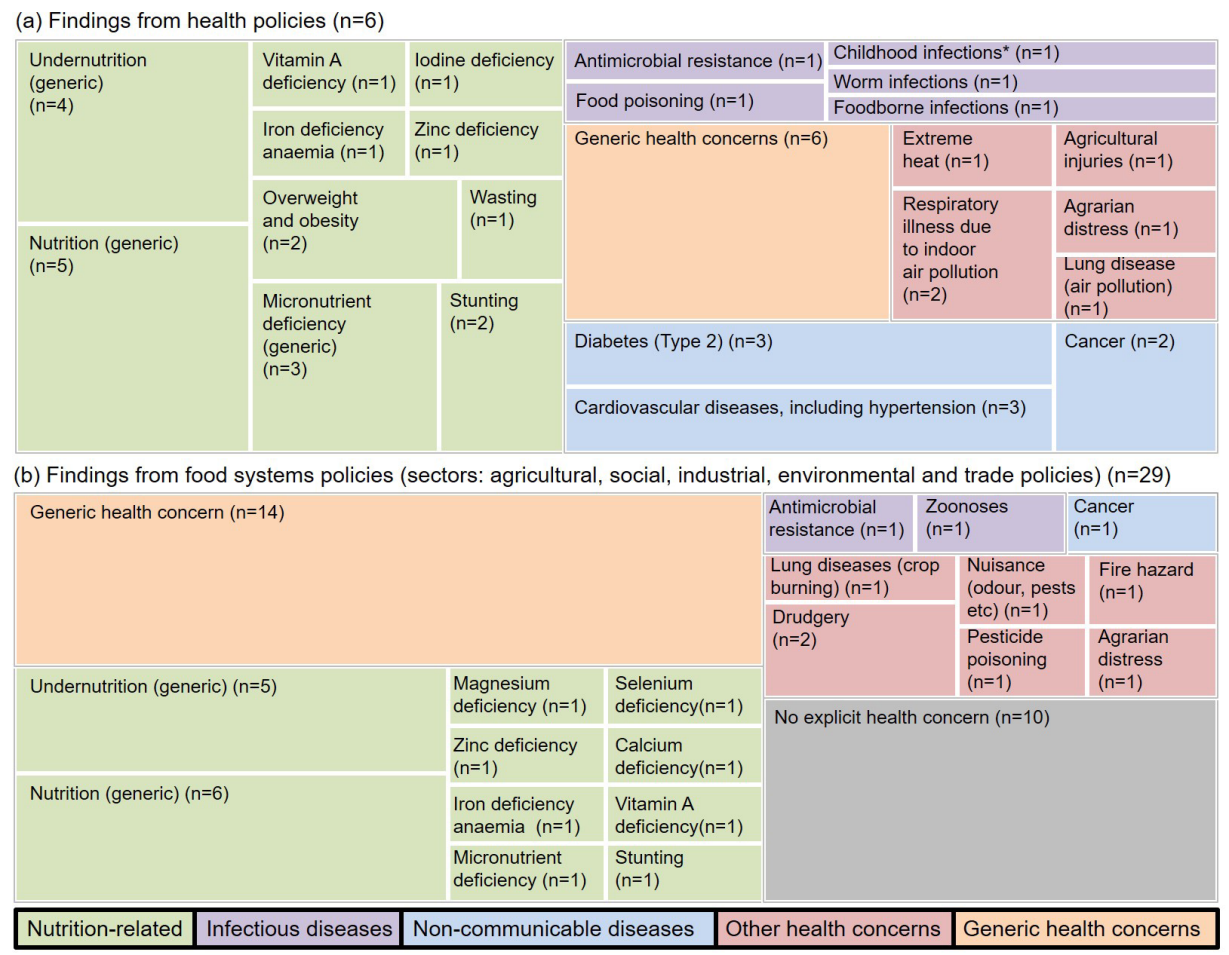
Food Systems-Related Health Concerns Mentioned in (a) Health Policies (n = 6); and (b) Food Systems Policies/Policies of Other Sectors (n = 29). Box size proportional to number of policies mentioning the respective health concern; “generic health concerns” indicates that ‘health’ or ‘well-being’ was explicitly mentioned; *includes diarrhoeal diseases. (Figure prepared using the *portfolio* package in R statistical software version 3.5.1 and edited in MS PowerPoint 2010).

Figure 1 depicts the prominence of nutritional concerns among all relevant health concerns in the analysed policies. Only one agricultural policy (NFS, which was just a pilot scheme of limited geographic and temporal scope) was designed primarily to address undernutrition (other agricultural policies were oriented towards productivity and remuneration). Some policies mentioned “nutritional security” (rather than nutrition) as a concern, relating to the availability of adequate nutritious food at country or household level (MIDH, NFS, NFSA, NFSM, NLM, NPF, and RKVY).

*“Main objectives of scheme are…. enhancing production and productivity, improve nutritional security and income support to farmers” *(RKVY).

 From a social security perspective, several population groups were indicated as vulnerable: (*i*) the landless and marginal farmers (ie, those owning under two acres of land); (*ii*) artisans; (*iii*) slum dwellers; (*iv*) informal sector workers; (*v*) the destitute; (*vi*) households headed by widows, the terminally ill, disabled, or elderly (ARMPD); (*vii*) HIV positive individuals (ARMPD and NPF); (*viii*) pregnant and lactating women; (*ix*) adolescent girls; (*x*) children (NFSA); (*xi*) those living in beggar homes; (*xii*) hostels with students from marginalised communities (ARMPD); (*xiii*) living in remote settings, including hilly and tribal areas (NFSA); and (*xiv*) communities facing shocks such as agrarian distress, seasonal migration, disruptions in access to services, disease outbreaks, endemic disease areas, and natural disasters (NNS and PDS). The only mention of “caste” was in the context of ensuring representation of scheduled castes in vigilance committees of the NFSA. In effect, various criteria such as socioeconomic, demographic, gender, shocks, caste, and health were used to identify beneficiaries or vulnerable groups, covering large section of the total population.

 While health policies have acknowledged these multiple vulnerabilities to undernutrition (ICDS, NHP, and NNS), their focus was on vulnerability of women, adolescent girls, and children to undernutrition and micronutrient deficiency (ICDS and NNS), and the strong gender and inter-generational dimension to the problem (NNS).

*“The girl child goes on to become an undernourished and anaemic adolescent girl, often deprived of … nutritional support, educational opportunities, … married too early, with early child bearing and inadequate inter-pregnancy recoupment. This perpetuates a vicious cycle of undernutrition and morbidity…” *(NNS).

 Women and children from scheduled castes, scheduled tribes, and particularly vulnerable tribal groups (these categories have been officially defined) were indicated as especially vulnerable (NNS), additionally in the context of climate change (NAPCCHH). Therefore, considerations for gender, demography, caste, and remoteness of communities were acknowledged. In effect, the population addressed by nutritional and health policies and programmes is a sub-set of the population recognised as food insecure. Agricultural, industrial, or trade policies did not include any explicit considerations on nutritional vulnerability.

 Subsidised foodgrains and pulses has been the main approach towards ensuring household food security, covering priority households and those under the *Antyodaya Anna Yojana *(a scheme for the poorest sections). Support is based on the number of members in the eligible household (NFSA). The food supplementation approach for women and children used by the health sector is a more targeted version of the broader food welfare approach. Nutritional supplementation is provided to pregnant and lactating women through the local *anganwadi* (crèche; ICDS and NFSA). Schemes for improving nutrition of adolescent girls through additional foodgrains were also introduced (ARMPD). For children, meals are provided at *anganwadis* or government schools free-of-charge. In addition, state governments have been directed to identify and provide meals to malnourished children based on prescribed standards (ICDS and NFSA).

*“Every child has a right to adequate nutrition and to be safeguarded against hunger, deprivation and malnutrition” *(NPC).

 In remote tribal areas, grants for setting up village grain banks were provided in some areas to store one quintal of grain per family to protect children in times of scarcity.

*“As a part of the Government’s efforts to prevent deaths of children in remote and backward tribal areas due to malnutrition, a Village Grain Banks scheme was launched in 1996-1997” *(PDS).

 In addition to food supplementation, the health sector also screens and treats severe nutritional deficiencies within the target groups. Pregnant women are monitored for weight gain (ICDS), screened for anaemia and managed accordingly (NHP). Screening for other micronutrient deficiencies was recommended (NHP). Support was also encouraged for enhanced care during and after illness for severely and acutely undernourished children (ICDS and NNS). Proposed steps towards this also include a second *anganwadi* worker for childcare and nutrition counselling through home visits (ICDS).

 Additionally, broader interventions such as female literacy, women’s empowerment and autonomy in decision-making and use of resources have been listed as critical towards improved maternal and child nutrition (NNS). Engaging vulnerable communities in decision-making, seasonal nutritional screening, and scaling-up nutrition action were recommended (NAPCCHH). Only one agricultural policy mentioned nutritional support, through the development of backyard poultry among “below poverty line” families, for whom livestock rearing was identified as an important intervention for livelihood (NLM).

###  The Focus on Non-communicable Diseases

 NCDs, while recognised as a major problem, were only mentioned in few health policies (NHP, NNS, and NPCDCS) and none of the food systems policies. The interventions to tackle these challenges were largely biomedical.

 NCDs were addressed in some health policies, with particular emphasis on obesity, CVD, diabetes, hypertension, stroke, and cancer (NHP and NPCDCS). Two health policies mentioned overweight and obesity (NNS and NPCDCS), with one policy identifying obesity as being especially prevalent among urban, wealthier, and older adults, and also discussed childhood obesity as a concern indicating a double burden of malnutrition in the country (NNS). Reduction of premature mortality due to CVDs, cancer, and diabetes by 25% by 2025 was listed as a priority (NHP). However, they were not listed as cross-sectoral goals, unlike stunting (NHP), despite unhealthy diet being a major risk factor for NCDs. Though the risk of NCDs is largely determined by circumstances created by non-health sectors, the analysed policies of those sectors conspicuously make no mention of NCDs.

 “*Non-communicable diseases are expensive to treat. National strategies have to focus on prevention and health promotion as key to reduce disease burden. Health education programme that promote exercise, weight reduction, early diagnosis, screening are some of the key interventions that need to be promoted at various levels of health facilities*” (NPCDCS).

 Interventions for those vulnerable to, or affected by, hypertension and diabetes, were primarily biomedical and operationalised through the healthcare sector under the aegis of the National Health Mission in urban and rural areas (NHP). Early detection has been emphasised (NHP), and screening of blood pressure and sugar has been supported from the primary care level (ie, sub-health centres) through auxiliary nurse midwives (ANMs), along with a referral system. “NCD clinics” were planned at sub-district and district hospitals (NPCDCS). Health promotion and counselling for behaviour change has been encouraged at all levels from sub-centre to district hospital, about diet and lifestyle management (NPCDCS). No specific interventions to prevent or control NCDs were found in agricultural, social, industrial, trade, or environmental policies.

###  Supporting Healthy and Balanced Diets

 Various policies have mentioned healthy and balanced diets in the context of (*i*) health implications of various food groups (ARMPD, NNS, NPCDCS 2013, PDS, and VIUC); (*ii*) dietary diversity (NHP, NFS, NNS, and NPF); and (*iii*) food safety (FSSA, NAPCCHH, and NMFP). Several interventions for supporting healthier diets were discussed: (*i*) food supplementation (ARMPD, ICDS, NHP, and NNS); (*ii*) fortification (ARMPD, NFS, NHP, and NNS); (*iii*) health promotion (ICDS, NAPCCHH, NNS, and NPCDCS); and (*iv*) regulatory and fiscal instruments (FSSA, FDIP, MEFS, MIDH, NFSA, NFSM, NHP, NNS, and NPF).

 Amongst various food groups, vegetables (VIUC), edible oils and fats (ARMPD) have been explicitly mentioned as important towards a balanced diet. Other food groups listed as (or implied to be) contributing towards nutrition included fruits, milk, eggs (NNS), coarse grains (NPF), and meat (PDS). However, there was inadequate coherent and nuanced articulation on healthy diets. Only the nutrition policy explicitly referred to a dietary guideline (ie, Nutrient Requirements and Recommended Dietary Allowances for Indians, 2010) for informing food supplementation programmes (NNS). The health policy identified healthy diets as a priority area, but offered no further details.

 “*The policy identifies coordinated action on …priority areas for improving … health:… balanced, healthy diets…*” (NHP).

 Diet has been recognised as a main risk factor for both micronutrient deficiency (NFS) and NCDs (NPCDCS). Relatively high intake of staples, such as wheat and rice, and low intake of fruits, vegetables, and animal products were associated concerns (NFS). Food groups of concern towards NCDs were:

 “*Unhealthy diet including high intake of salt, sugar and trans-fats and low intake of fruits and vegetables*” (NPCDCS 2013).

 Improving dietary diversity was identified as contributing to a healthy diet (NHP, NFS, and NNS) and as an avenue for “coordinated action” (NHP). However, it was seen as not feasible from a short-term perspective.

 “*While dietary diversification remains the most desirable way forward, supplementation and fortification require to be considered as short- and medium-term solutions to fill nutrient gaps*” (NHP).

 Standards for fortification of wheat flour and oil have been prepared (ARMPD). The use of iron and iodine-fortified salt has been recommended (NNS). The nutrition policy has encouraged other ministries to promote local processing of nutritious foods through training women’s groups, strengthening supply systems (for vegetables and animal products), and improve quality of mid-day meals for children (NNS).

 “*Use SSA (universal education programme ) flexi funds for kitchen gardens in/around school premises contributing to addition of local/seasonal vegetables and fruits…*” (Recommendation to Ministry of Human Resource Development in NNS).

 The health sector has also used health promotion through awareness for addressing undernutrition (ICDS) and NCDs (NPCDCS). The engagement of local mothers of well-nourished children through positive-deviance-hearth approach was suggested to help mothers optimally utilise local resources for higher quality diets (NNS). State NCD cells were tasked with the responsibility to make the public aware about dietary risk factors and healthy foods. Folk media has been suggested to reach rural and urban poor communities (NPCDCS). ANMs were also expected to create awareness about healthy foods (NPCDCS). Climate change, nutrition, and health was also planned to be incorporated into school and college curricula (NAPCCHH).

 A role for Indian Systems of Medicine (AYUSH) in health promotion for lifestyle modification was also recognised (NHP). Some of these systems have a substantial focus on dietary interventions. However, this was not described in further detail.

 “*Promotion of healthy living and prevention strategies from AYUSH systems… has a special appeal and acceptability in the Indian context*” (NHP).

 A regulatory approach has been in place to ensure that food products produced by manufacturers, and claims made about food products by manufacturers or advertisers are in accordance with standards that safeguard health (FSSA).

 “*… endeavour to achieve an appropriate level of protection of human life and health and the protection of consumer’s interests…with reference to food safety standards and practices*” (FSSA).

 Several regulatory and fiscal instruments have also been used to control prices of staple, nutritionally important foods such as pulses (MEFS), with recommendation to also cover milk, eggs, fruits, and vegetables (NNS). Regulations have also been made in the context of foreign direct investment in food retail, agriculture, livestock, and aquaculture (FDIP) but there was no explicit health consideration. It was also suggested that fiscal instruments could be considered to modify behaviour, but no further details were provided on commodities to be targeted.

 “*The Government could consider imposing taxes on specific commodities - such as … foods having negative impact on health…*” (NHP).

 The inclusion of “nutritious millets” in the food security programme was recommended (NPF). Bio-fortified food crops were identified and promoted in a pilot scheme (NFS). Agricultural policies also supported horticulture (fruits, vegetables, nuts, and mushrooms) (MIDH), and encouraged backyard poultry among poor families (NLM). To promote access of vegetables in urban areas, production through cluster approach was also supported (VIUC). Year-round availability of certain fresh produce is an emerging demand (RKVY), with potential negative implications for seasonal foods. The promotion of several micronutrient-rich varieties of staples, sweet potato, and drumstick (*Moringa oleifera*) and effective supply to target communities was also tried in a pilot scheme (NFS).

###  Addressing Health Concerns Beyond Nutrition and NCDs

 Nutrition-related concerns were not the only food systems-related health concerns in the reviewed policies (Figure 1). However, only few policies explicitly named specific concerns: (*i*) food production-related health concerns included antibiotic resistance (NHP), agricultural injuries (MIDH, NHP, NMAET, and NPF), agrarian distress (NPF, NNS and implied in ADWDRS, ESC, MNREGS, NAPCCHH, and RKVY), and pesticide poisoning (PMB); (*ii*) consumption-related health concerns (besides undernutrition and NCDs) include food poisoning (FSSA) and food-borne infections (NAPCCHH); and (*iii*) waste management-related health concerns included various hazards associated with crop waste (NPMCR) and food waste (NMFP, NMSA, RKVY, and SWMR). Concerns related to new technologies were also discussed (BDA, FSSA, FTP, NLM, NPF, PMB, and PPVFRA). Few documents only mentioned a generic concern for health (ARMPD, BDA, FDIP, FTP, MNREGS, and PPVFRA), for instance:

 “*Safety and health norms applicable to domestically produced goods shall apply, mutatis mutandis, to imports, unless specifically exempted*” (FTP).

 To address antimicrobial resistance, strengthening pharmacovigilance against the use of antimicrobial agents as growth promoters in livestock was recommended (NHP). However, this was not discussed in the livestock policy, where the focus was more on vaccinations, animal health, and other biosecurity threats (NLM).

 A common concern across health and agricultural policies was to reduce agricultural occupational injuries (to half by 2020 (NHP)), and this was identified as a cross-sectoral goal (NHP). Towards this, agricultural policies have supported mechanisation and better-adapted instruments as key interventions, especially to reduce drudgery among women (MIDH, NMAET, and NPF).

 “*Horticulture mechanization is aimed to improve farm efficiency and reduce drudgery of farm work force*” (MIDH).

 Further mitigation of agrarian distress was recommended (NPF). The categories of farmers identified as vulnerable included tribal farmers, pastoralists (issues of access to common lands, grazing, camping rights, and migration paths), plantation farmers (market fluctuations in price), and island farmers (transportation costs and natural disasters) (NPF). There was cognisance about the potential impact of climate change, which was expected to “reduce farm incomes by up to 20-25 percent in the medium term” (ESCB and NAPCCHH). Awareness campaigns about debt waiver schemes (ADWDR), farming insurance schemes (NPF and MIDH), and general extension services towards various schemes (RKVY) are planned or recommended. Assistance has been provided for promoting farmer-interest groups and farmer-producer organisations, which may strengthen their bargaining position, make production more efficient, and also reduce risk (MIDH).

 Heat stress was also mentioned as a concern for farmers (NAPCCHH), and the employment scheme recommended that shade, drinking water, and childcare facilities be provided at the worksite (MNREGS). Occupational health of workers of pesticide manufacturing plants and of farmers using pesticides was of concern. Regular medical examination of pesticide industry workers was recommended (PMB). Management of misbranded or damaged pesticides were also discussed, alongside the mandated notification of cases of pesticide poisoning (PMB).

 Various agricultural policies supported or encouraged progressively shifting to eco-friendly technologies, conservation of natural resources, location-specific agronomic practices, judicious use of chemicals, crop diversification, and adoption of crop-livestock farming systems (MIDH, NMAET, NMOOP, NMSA, NFSM, NPF, and WSD). While these may have an important bearing on health, it was not explicitly discussed in these policies.

 Food-borne infections were mentioned in the context of climate change, with recommendations for additional capacity in food safety and nutrition monitoring (NAPCCHH). Food poisoning cases are notifiable, to enhance food safety (FSSA).

 Another important concern is indoor air pollution from unclean fuels in kitchens (NAPCCHH and NHP), but this topic is beyond the scope of this paper. To alleviate health concerns raised by air pollution and carcinogens due to the prevalent crop residue-burning practices, various approaches were suggested, such as through mulching or use in industry (NPMCR). The waste management rules aimed to minimise fire hazard and nuisance of rodents, flies, birds and animals at landfills (SWM), all of which are related to food waste. Moreover, equipment, including gloves, coats, masks, and footwear were required be provided to handlers of solid waste (SWM).

 “.*.. minimise impact of solid waste on human health and environment*” (SWM).

 Agricultural policies have discussed biosecurity threats through import of pigs (NLM) and birds (NPF), which may carry disease-causing agents. The approaches adopted to address this include regulation of import and facilities for quarantine. Policies also mentioned human health-related concerns or discussed approaches to regulate new food products (FSSA), new pesticides (PMB), living modified organisms (BDA), plant varieties with restriction use and “terminator technology” (PPVFR), and imported products (FTP). It was stated that genetic technology also needs to be assessed for risks and benefits (BDA and NPF).

 “*…to regulate, manage or control the risks associated with … living modified organisms … likely to have adverse impact on the conservation and sustainable use of biological diversity and human health*” (BDA).

###  Involvement of Health Ministries in Food Systems Governance

 Intersectoral coordination for health in food systems governance was found to have occurred in two ways: (*i*) health policies encouraging other ministries towards nutrition-sensitive planning; and (*ii*) inclusion of health ministry representatives in inter-ministerial bodies.

 “*The policy articulates to institutionalise inter-sectoral coordination at national and sub-national levels to optimise health outcomes, through constitution of bodies that have representation from relevant non-health ministries … in line with the emergent international “Health in All” approach as complement to Health for All*” (NHP).

**Figure 2:**
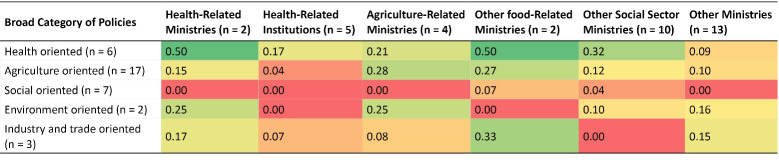
Mentions of Various Ministries and Health Institutions Within Each Policy-Category. Green indicates relatively higher proportion of policies mentioning the respective category of ministries; red indicates no mentions; calculated as follows: (number of policies mentioning respective ministries)/(total number of respective policies * total number of respective ministries); prepared in MS Excel 2010.

 Such articulation for inter-sectoral coordination was stronger and more specific for undernutrition, where the role of the MoHFW has been proposed as convenor of inter-sectoral action (NHP and NNS). Addressing stunting has been mentioned as a “cross-sectoral goal.” Health policies mentioned other ministries more often as compared to food systems policies (Figure 2).

 “*A wide spectrum of national programmes contribute to improved nutrition outcomes…These include the Integrated Child Development Services…Targeted Public Distribution System, National Food Security Mission, Mahatma Gandhi National Rural Employment Guarantee Scheme…among others*” (NNS).

 “*Policy envisages that the MoHFW would take on the role of convener to monitor and ensure effective integration of both nutrition-sensitive and nutrition-specific interventions for coordinated optimal results*” (NHP).

 While “convergence with other related departments” was also mentioned in the NCDs policy document (NPCDCS), it is unclear whether it meant convergence of departments within the health ministry or outside it, and what the objective of this convergence was. The nutrition policy has provided specific suggestions for agricultural and other sectors to focus on food and nutritional security of vulnerable groups (NNS).

 “*Promote processing of locally available nutritious foods through training of women’s (groups)… and use this for nutrition communication*” (suggestion to the Ministry of Food Processing Industries in NNS).

 For the priority concern of maternal and child undernutrition, leadership was under Ministry of Women and Child Development, with the MoHFW as a key partner in all the envisaged programmes (ICDS and NNS). The interventions go beyond biomedical approaches, through convergence with other ministries, such as public distribution and agriculture at the level of the *anganwadi* (ICDS).

**Figure 3:**
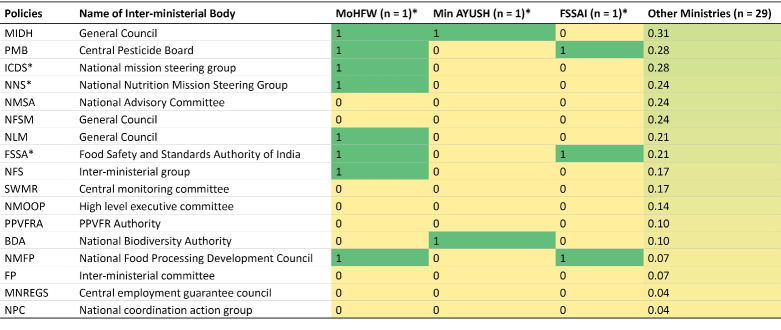
Inclusion of Health Ministry Representatives in Inter-ministerial Bodies Governing Food Systems in India. *Health-oriented policy or ministry/institution; green indicates inclusion; yellow indicates not included; calculated as follows: (total number of ministries mentioned)/(total number of ministries in the category); prepared in MS Excel 2010. Abbreviations: BDA, Biological Diversity Act; FP, Fertilizer Policy; FSSA, Food Safety and Standards Act; ICDS, Integrated Child Development Services; MIDH, Mission for Integrated Development of Horticulture; Min AYUSH, Ministry of Indian Systems of Medicine; MNREGS, Mahatma Gandhi National Rural Employment Guarantee Scheme; MoHFW, Ministry of Health and Family Welfare; NFS, Nutri-farms Scheme; NFSM, National Food Security Mission; NLM, National Livestock Mission; NMFP, National Mission on Food Processing; NMOOP, National Mission on Oilseed and Oil Palm; NMSA, National Mission on Sustainable Agriculture; NNS, National Nutrition Strategy; NPC, National Policy for Children; PMB, Pesticide Management Bill; PPVFRA, Protection of Plant Varieties and Farmers Rights Act; SWMR, Solid Waste Management Rules.

 Food systems policies either engaged MoHFW representatives in their respective inter-ministerial advisory or steering groups, or involved health institutions such as the Food Safety and Standards Authority of India (FSSAI) and the Indian Council of Medical Research (ICMR) (Figure 3). For example, as part of the ‘General Council’ instituted by the horticulture mission, representation from the MoHFW and the Ministry of AYUSH (Indian systems of medicine) were included (MIDH) (Figure 3).

 “*General Council will be the formulation body giving overall direction and guidance to Mission, monitor and review its progress and performance”* (MIDH).

 Of the 35 analysed policy documents, 17 had instituted inter-ministerial steering or executive groups. Of these, nine had involvement of a health-related ministry (Figure 3). Of these nine groups, two were regulatory (FSSA and PMB, which are related to food and worker safety) enforcing rules to safeguard health, five were advisory and oversight-related (BDA, MIDH, NFS, NLM, and NMFP, which are mainly related to food production and processing) towards policy advice and review, and two were action-oriented (ICDS and NNS, related to addressing undernutrition), which were aimed at coordinating the implementation of programmes. In food systems policies, the MoHFW found itself mainly in advisory groups. No further specifications on the role of the involved ministries were provided in these documents. Representatives of health ministries in most of these groups were those with political or bureaucratic background rather than health expertise. Some groups also involved the Indian Council of Medical Research (NAPCCHH and PMB) and the National Institute of Nutrition (PMB) in a governance role.

 Among the analysed policies, the nutrition policy cross-referenced policies from agricultural and social sectors (NNS), whereas policy documents on agricultural programmes and missions only cross-referenced other agricultural policies (Supplementary file 1, Table S2). Barring some minor additional sources, most of the agricultural (MIDH, NFS, NLM, NMOOP, NMFP, NMSA, RKVY, and WSD), social (MNREGS and NFSA), environmental (NPMCR), and health (ICDS, NAPCCHH, and NPCDCS) schemes are funded by the central and state governments (with the centre providing the majority share).

## Discussion

 The findings have been discussed in three sections, namely (*i*) nutrition (covering themes 1, 2, and 3 pertaining to undernutrition, NCDs, and healthy diets); (*ii*) other health concerns (covering theme 4); and (*iii*) intersectoral coordination (covering theme 5). Some of our inferences used the Health in All Policies paradigm, to consolidate our arguments on health considerations in food systems policies.

###  Nutrition

 Undernutrition received relatively high policy attention. Literature affirms that there has been a strong degree of political will for addressing undernutrition in India for several decades,^25^ and this continues to exist.^4,28^ However, the NNS is by no means a comprehensive “nutrition” strategy because NCDs and their risk factors (eg, ultra-processed foods) have not been included. The disproportionate policy attention to undernutrition may be because it is an older problem, has relatively higher burden, and hunger is deemed unacceptable.^78^

 In contrast, NCDs were addressed only by health policies, for which only biomedical solutions have been instituted. This neglect of NCDs in food policy is important due to the burden of NCDs on families and the health system, and also the potential for prevention. This may be explained by the larger neglect of social and commercial determinants of health, which include, among others, the strong influence of corporations on food environments and consequently on NCDs.^79-82^ The gap in health consideration in trade policies is also related to similar factors, such as the primary objective of trade policies being economic development, neoliberal ideology, and power disparities between actors,^83^ and shifting the health responsibility to the consumer.^84^ These aspects have also been reflected in multi-country trade deals involving high-income countries.^85^ The challenge is further explainable from the continuing difficulties of health consideration even in tobacco trade policies.^86,87^ On a similar line, “counterproductive policies,” such as those increasing access to highly processed foods, were also of concern.^4^ Empirical insights on mechanisms through which food trade may impact health are available,^88,89^ and there are also calls for specifically improving coherence between trade policies and nutrition goals.^90^ We found some coherence in identification and targeting of vulnerable groups for undernutrition, but not for other challenges. Recent literature has described policy opportunities from the perspective of policy actors to tackle the double burden of malnutrition in India.^4^

 Our research also showed that dietary guidelines were ignored by food systems policies. There are at least two guidelines developed in India on healthy diets. The NNS was the only policy document that mentioned one of them,^28^ and the other guideline was *Dietary guidelines for Indians*, put forth by the National Institute of Nutrition.^91^ The latter claims that it could be considered towards reaching “the goals specified in the National Nutrition Policy.”^91^

 The nutri-farm scheme was the only agricultural policy explicitly addressing undernutrition (NFS). However, while the objective of the NFS was to improve dietary diversity, it surprisingly focused predominantly on cereals. This may reflect the perception of fortified cereals as a ‘silver-bullet’ for undernutrition, and also the preoccupation with production and food security. A few other policies, such as the livestock and horticulture missions, have potential for stronger nutritional thrust, as was recognised for the latter in an earlier study.^4^

###  Other Health Concerns

 Concerns related to agricultural injuries, antibiotic resistance, food safety, crop-residue burning, and food waste disposal were identified. With the majority of India’s working population dependent on agriculture,^92^ designating agricultural injuries as a priority concern was understandable. However, aspects related to mental^93^ and social well-being of agrarian households^94^ was not adequately addressed. For instance, there was no mention of farmer suicides. As these challenges are driven by agricultural and economic policies, they may be most efficiently dealt with at that level.

 Health impacts due to crop-residue burning^95^ has recently started receiving attention from the public health sector.^96^ Avian flu and other zoonoses were inadequately discussed in the included policy documents, but health system measures to address these for humans^97^ and animals^98^ were in place. While antibiotic resistance due to use in livestock production was mentioned as a concern in the health policy, it was missing from the livestock mission, demonstrating another gap in coherence. Overall, the impacts of food system policies on various health risks and outcomes (whether positive or negative) are potentially incidental rather than planned.

###  Intersectoral Coordination

 The NNS and the NHP emphasised intersectoral coordination to address undernutrition, possibly also because they were released in 2017 after the launch of the 2030 Agenda for Sustainable Development. A cautionary note is that similar calls for intersectoral coordination were made in the 1993 National Nutrition Policy, but those aspirations were not adequately realised.^25^ From the NHP, it appeared that the health ministry felt it within their power to employ nutrition-specific interventions such as food supplementation, but beyond their immediate power to address dietary diversity.

 The NHP recognised the potential of inter-ministerial bodies “to optimise health outcomes,” bearing relevance for operationalising “Health in All.” Literature suggested that there were limitations to positing health as the main argument for developmental planning and interventions,^21^ and hence, involvement of the health ministry in “advisory” roles in many of these groups may be an adequate starting point. The impact of this involvement would have to be studied through different data.

 Health considerations in food policy may also be more challenging due to the complex nature of causal linkages, as compared to, for example, asbestos regulation, for which the health basis for policy intervention is straightforward.^99^ To foster healthier food systems, there were suggestions to improve the understanding about nutrition among agricultural policy-makers,^100^ linking policy agendas across sectors,^34^ advocacy,^4^ and also build relationship between food systems actors,^101^ which the nutrition policy (NNS) has shown some intention of doing. Another recommendation was for improved data from the health and agricultural sector to provide feedback on the impact of policy interventions,^34^ but approaches to strengthen data systems were only discussed in passing in the health policies (NNS and NHP). The ICDS scheme is an example of a programme incorporating nutritional and other goals at upstream planning level with downstream coordinated implementation at creches.

###  General Contributions

 To our knowledge, this is the first paper that considered a broad range of health outcomes while studying food systems policies, in contrast to the usual focus on undernutrition and NCDs. Our method helped navigate and weave together diverse findings from a range of sectors. The empirical findings on the policy focus on undernutrition may resonate with other low- and middle-income countries, but those on the involvement of the health ministry in food systems governance may be more widely relevant. Various typologies were offered through the study: (*i*) factors contributing towards vulnerability to undernutrition; (*ii*) responses towards undernutrition; (*iii*) topics pertaining to healthy and balanced diets; (*iv*) non-nutritional health concerns of food systems policies; (*v*) broad approaches to intersectoral coordination for health; and (*vi*) specific types of involvement of the health ministry in food systems governance. Each of these may be of value for research in other settings and/or for policy analyses of other sectors.

###  Limitations

 The focus of the analysis was limited to national level policy. The Constitution of India has divided responsibilities related to food systems between national and state governments. For example, international trade and establishment of food standards are on the national list; agriculture, animal husbandry, irrigation, land, and markets are on the state list; and social security and trade in food stuff are on the concurrent list.^102^ Hence, a better view of the policy landscape could be achieved by analysing policy content from one or more states in India in addition to what has been done here. For example, irrigation, a state subject, has several associated health risks, such as VBDs.^10^ However, national-level policy has considerable influence on local action,^103^ and most of the funding for food systems-related programmes stems from the national government, and so it was worthwhile focusing on national policies to start with.

 A richer picture on operationalisation and practice of policies would emerge through key informant interviews in addition to document analysis. Furthermore, some food-group specific policies, which may have had important implications for health have not been included, for example the so-called “beef ban” policy. There was no clear policy document, and there were state-level variations on how these have been implemented. However, such policies have important repercussions on health, especially for marginalised groups.^104^

## Conclusion

 In food systems policy in India, undernutrition was considered to some extent, NCDs were completely ignored, and details on other health concerns were often superficial. Some evidence of intersectoral coordination and policy coherence were found for the management of undernutrition, but not for any other health outcomes. Policies spearheaded by the health ministry for nutrition-specific interventions, such as the ICDS, best exemplified ideas and actions for intersectoral coordination for better health outcomes. The participation of health ministry representatives in few advisory groups of food production and processing policies theoretically provided an avenue for fostering health considerations in those areas, but such groups were not found for the included social and trade policies. Policies were understandably focused on sectoral objectives, including productivity, economic development, technology or environmental protection. Gaps from the health perspective warrant further policy attention to help address the double burden of malnutrition, and also the several other relevant health concerns.

 The coronavirus disease 2019 (COVID-19) pandemic further highlighted the vulnerabilities of food systems and their health ramifications in India and elsewhere,^105,106^ emphasising the need for broad analyses, as was presented here. Our study contributed potential methods and typologies for such analyses. Our findings also set the stage for further studies on specific health concerns, specific inter-ministerial and policy interlinkages, and also similar research in other development sectors.

## Ethical issues

 This study obtained ethical approval from the Padmashree Institute of Clinical Research in Bengaluru, India (reference no. IEC-BIO-004; approval date: 10 August 2018) and the Ethics Commission of Northwestern and Central Switzerland (EKNZ, reference no. BASEC Nr Req-2018-00839, approval date: 19 October 2018). No data were collected from people or animals.

## Competing interests

 Authors declare that they have no competing interests.

## Authors’ contributions

 Conceptualisation: AP; supervision: AM, JU, and MSW; data collection and analysis: AP; review of analysis: AM and MSW; preparation of first draft: AP; review of drafts: AM, JU, and MSW.

## Funding

 No specific funding was received for the study. The first author is a PhD student from India on a Swiss Government Excellence Scholarship (ESKAS), which covers his fees and living costs. ESKAS did not have any role in the design and execution of the study and the decision to submit for publication.

## Supplementary files

Supplementary file 1 contains Tables S1- S2.
