# Supplementary material for: Health in Food Systems Policies in India: A Document Review
Source: Int J Health Policy Manag. 2021 Mar 15;11(7):1158–71. doi: 10.34172/ijhpm.2021.18 (PMC9808200; doi:10.34172/ijhpm.2021.18)
Supplement: Supplementary file 1 — contains Tables S1- S2. [file ijhpm-11-1158-s001.pdf]

**Article title:** Health in Food Systems Policies in India: A Document Review

**Journal name:** International Journal of Health Policy and Management (IJHPM)

**Authors' information:** Adithya Pradyumna[1,2,3]*, Arima Mishra[3], Jürg Utzinger[1,2], Mirko S. Winkler[1,2]

[1]Swiss Tropical and Public Health Institute, Basel, Switzerland.

[2]University of Basel, Basel, Switzerland.

[3]Azim Premji University, Bengaluru, India.

(*corresponding authors: adithya.pradyumna@apu.edu.in)

**Supplementary file 1**

**Table S1.** Coding Scheme

| **Themes** | **Categories** |
|---|---|
| Undernutrition recognised and addressed as cross-sectoral concern | Under-nutrition related, nutrition security, obesity related; vulnerable groups, household food security, food security, food affordability, health systems responses to undernutrition |
| The focus on NCDs | Chronic-disease related, health systems responses to NCDs |
| Supporting healthy and balanced diets | Food groups, food diversity, food safety, food supplementation, food fortification, health promotion, regulatory instruments, fiscal instruments |
| Addressing health concerns beyond nutrition and NCDs | Other health problems, generic health concerns, environmental sustainability, animal health, trade and business, other health determinants |
| Involvement of health ministries and institutions in food systems governance | Health sector as lead agency, inter-ministerial groups involving health sector, inter-ministerial groups without involving health sector, health being encouraged in other sector action, health through intersectoral action, resources for action |

**Table S2.** Cross-References by Policies to Other Policies in the Sample

| Policies | RKVY | MNREGS | MIDH 2014 | NFSM | FSSA 2006* | NMSA 2014 | VIUC 2011 | CPDS 10FYP | FTP 2015 | NLM 2016 | NMOOP 2014 | NNS 2017* | BDA 2002 | FP 2015 | ICDS * | NFSA 2013 | NHP 2017* | NMAET 2012 | NMFP 2013 | NPC 2013 | NPF 2007 | PMB 2017 | PPVFRA 2001 |
|---|---|---|---|---|---|---|---|---|---|---|---|---|---|---|---|---|---|---|---|---|---|---|---|
| NNS 2017* | 1 | 1 | 1 | 1 | | | | 1 | | | | 1 | | | 1 | 1 | 1 | | | 1 | | | |
| NMSA 2014 | 1 | 1 | 1 | 1 | | | 1 | | | 1 | 1 | | | | | | | 1 | 1 | | | | |
| NPF 2007 | 1 | 1 | 1 | 1 | | | | | | | 1 | | 1 | 1 | | | | | | | | | 1 |
| MIDH 2014 | 1 | 1 | | | | 1 | 1 | | | | | | | | | | | | | | | | |
| RKVY | | 1 | 1 | 1 | | | 1 | | | | | | | | | | | | | | | | |
| NLM 2016 | 1 | 1 | | | | | | | | 1 | | | | | | | | | | | | | |
| FSSA 2006* | | | | | | | | | 1 | | | | | | | | | | | | | 1 | |
| NFS 2013 | 1 | | | | | | | | | | | 1 | | | | | | | | | | | |
| VIUC 2011 | 1 | | 1 | | | | | | | | | | | | | | | | | | | | |
| FP 2015 | | | | | | | | | | | | | | | | | | | | | 1 | | |
| FTP 2015 | | | | | | | | | 1 | | | | | | | | | | | | | | |
| NAPCCHH 2016* | | | | | | 1 | | | | | | | | | | | | | | | | | |
| NFSA 2013 | | | | | | | | 1 | | | | | | | | | | | | | | | |
| NHP 2017* | | | | | 1 | | | | | | | | | | | | | | | | | | |
| NMFP 2013 | | | | | 1 | | | | | | | | | | | | | | | | | | |
| NMOOP 2014 | | | | | | 1 | | | | | | | | | | | | | | | | | |
| NPMCR 2014 | 1 | | | | | | | | | | | | | | | | | | | | | | |
| PMB 2017 | | | | | 1 | | | | | | | | | | | | | | | | | | |
| **Total policies citing this policy** | **8** | **6** | **5** | **4** | **3** | **3** | **3** | **2** | **2** | **2** | **2** | **2** | **1** | **1** | **1** | **1** | **1** | **1** | **1** | **1** | **1** | **1** | **1** |

Green indicates reference to current policy or scheme; red indicates reference to a service rather than the policy; and yellow indicates reference to an older policy in the same domain;*health-related policy document; Policies which were not cross-referenced by any other policies: FDIP, NAPCCHH, NFS, NPCDCS, NPMCR, SWMR; policies not cross-referencing any other policies from the sample: BDA, CPDS, FDIP, ICDS, MNREGS, NFSM, NMAET, NPC, NPCDCS, PPVFRA, SWMR; WSD cross-referenced MNREGS and MIDH, but was not included here due to lack of space
